# Carcinogenic adducts induce distinct DNA polymerase binding orientations

**DOI:** 10.1093/nar/gkt554

**Published:** 2013-06-28

**Authors:** Kyle B. Vrtis, Radoslaw P. Markiewicz, Louis J. Romano, David Rueda

**Affiliations:** ^1^Department of Chemistry, Wayne State University, Detroit, MI 48202, USA and ^2^Department of Medicine, Section of Virology, Imperial College London, London W12 0NN, UK

## Abstract

DNA polymerases must accurately replicate DNA to maintain genome integrity. Carcinogenic adducts, such as 2-aminofluorene (AF) and *N*-acetyl-2-aminofluorene (AAF), covalently bind DNA bases and promote mutagenesis near the adduct site. The mechanism by which carcinogenic adducts inhibit DNA synthesis and cause mutagenesis remains unclear. Here, we measure interactions between a DNA polymerase and carcinogenic DNA adducts in real-time by single-molecule fluorescence. We find the degree to which an adduct affects polymerase binding to the DNA depends on the adduct location with respect to the primer terminus, the adduct structure and the nucleotides present in the solution. Not only do the adducts influence the polymerase dwell time on the DNA but also its binding position and orientation. Finally, we have directly observed an adduct- and mismatch-induced intermediate state, which may be an obligatory step in the DNA polymerase proofreading mechanism.

## INTRODUCTION

**Figure 1.**
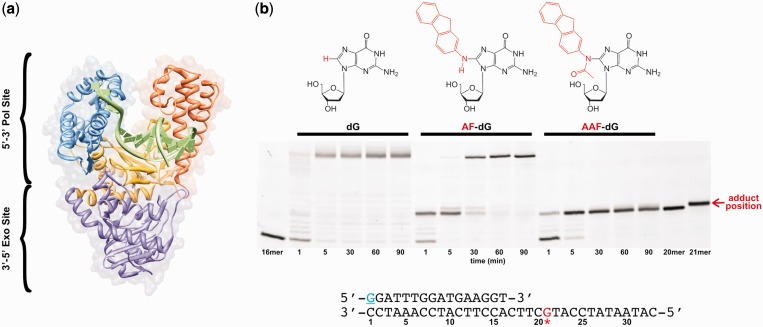
Carcinogenic adducts induce polymerase stalling on the DNA. (**a**) DNA polymerase structure. The fingers (blue), thumb (orange) and palm (yellow) domains encompass the pol site, where 5′–3′ template-directed DNA synthesis occurs. Misincorporated nucleotides can be excised at the exo site (purple), which increases the overall fidelity of the polymerase. Crystal structure from *B. stearothermophilus* DNA polymerase I (PDB ID 1l3s), a close structural homolog of KF. (**b**) Chemical structures of deoxyguanosine (dG), *N*-(deoxyguanosin-8-yl)-2-aminofluorene (AF-dG) and *N*-(deoxyguanosin-8-yl)-*N*-acetyl-2-aminofluorene (AAF-dG). Below the structures is a running start DNA polymerase extension assay for the extension of the primer–template shown below the gel. The primer has a 5′-Cy3 attached to the underlined, blue G. The template either has an unmodified dG, an AF-dG or an AAF-dG at position 21 (red G in template). Three different reactions were carried out with the indicated primer–templates, with aliquots being removed from the reaction mixture and stopped by addition of an equal volume of loading buffer (10 mM ethylenediaminetetraacetic acid, 1 mg/ml bromophenol blue, in 10 ml of formamide) at the indicated time points. The samples were run on a 20% denaturing polyacrylamide gel and scanned for Cy3 on a Typhoon 9210 Variable Mode Imager (GE Healthcare).

Most cancers propagate from an accumulation of mutations in genes that control cell growth (1,2). It is estimated that only 5–10% of cancer cases are attributed to inherited genetic defects, with the remaining cases resulting from lifestyle and environmental factors, such as chemical carcinogens (3). Arylamines are a well-studied class of carcinogen found in numerous occupational settings, tobacco smoke and chemical dyes (4–6). *N*-acetyl-2-aminofluorene is a potent, arylamine mutagen that after metabolic activation *in vivo*, forms two different adducts at the C8 position of guanine bases (Figure 1): 2-aminofluorene (AF-dG) and *N*-acetyl-2-aminofluorene (AAF-dG) (7–9).

Although AAF-dG and AF-dG adducts differ only by an acetyl group, the two adducts have different structure in duplex DNA (10–13). Although both adducts display considerable conformational heterogeneity, the major conformation of the AAF-dG in duplex DNA has the guanine rotated into a *syn* conformation with the AAF moiety intercalated into the DNA helix. Numerous solution structures of AF-dG adducts in several duplex sequence contexts indicate that in most sequences, the major structure is one where the AF resides in the major groove with the guanine in a normal *anti* orientation so that proper base paring can occur with a complementary cytosine.

It is thought that these structural differences lead to different effects on DNA synthesis and are thought to be related to the distinct mutation profiles that have been observed (14,15): AF-dG adducts cause high-fidelity polymerases to pause before bypass occurs (16,17) and primarily induce base substitution mutations (18), whereas AAF-dG adducts are strong blocks to DNA synthesis (17) and predominantly induce frameshift mutations, although base substitutions can also occur (15,18,19). Although structural studies of AF-dG adducts located at a primer–template junction (20) and positioned at the pre- and post-insertion sites in *Bacillus stearothermophilus* DNA polymerase I fragment (BF) (21) have provided some insight into the structures that might lead to misincorporation across from this adduct, the molecular mechanism and dynamic processes that lead to a mutation remain unclear.

DNA polymerases are structurally analogous to a right hand, complete with fingers, thumb and palm domains (pol site) (Figure 1a) (22). During 5′–3′ template-directed polymerization, the formation of a phosphodiester bond between the incoming deoxynucleoside 5′-triphosphate (dNTP) and the 3′-OH of the growing primer strand is catalyzed in the pol site. Additionally, most replicative polymerases are associated with a 3′–5′ proofreading exonuclease activity (23–25). This activity can be part of the polymerase itself (exo site) or be located on a separate subunit, but, most significantly, in each case it is positioned a significant distance away from the pol site (Figure 1a). As the name suggests, the role of the proofreading activity is to increase the accuracy of the polymerase by excising misincorporated nucleotides (23,26,27). DNA transfer between the pol and exo sites must be carefully regulated because unnecessary nucleotide excision from the growing DNA strand would needlessly slow DNA synthesis and consume essential chemical energy in the cell. The mechanism by which DNA polymerases transfer the DNA from the pol to the exo site remains elusive.

In the present study, we have used two powerful real-time single-molecule approaches to monitor the interactions between *Escherichia coli* DNA polymerase I (Klenow fragment, KF) and DNA primer-templates containing either an AF-dG or AAF-dG adduct. To help elucidate the mechanism by which these adducts affect DNA synthesis, we have determined the effects of these adducts on DNA polymerase binding to a primer-template in which the primer terminates either before or across from the adduct position. Our data show that either adduct linked to the template in the single-stranded DNA (ssDNA) region does not disrupt the binding orientation of the DNA polymerase. However, positioning either adduct on the templating guanine of the terminal base pair causes the polymerase to bind in two distinct orientations: one that is consistent with the primer positioned in the exo site and a second, previously unreported, intermediate orientation that is distinct from either pol or exo site binding. The presence of the next correct dNTP rescues pol site binding in the case of the AF-dG adduct, whereas this change is not observed for the AAF-dG adduct. This distinction possibly provides further evidence for why AAF-dG adducts cannot be bypassed by high-fidelity polymerases, whereas AF-dG adducts are bypassed after a brief stall at the adduct position. The intermediate orientation was also observed in the presence of a single mismatched primer, raising the intriguing possibility that this orientation represents a key intermediate in the polymerase proofreading mechanism.

## MATERIALS AND METHODS

### DNA constructs

All DNA oligonucleotides sequences are listed in Supplementary Table S1. All oligos were purchased from Eurofins MWG Operon. Oligos were purified by HPLC using a C18 column. AAF and AF-modified templates were prepared as previously described (28). Briefly, 20 nmol oligonucleotide containing a single guanine was incubated with 500 nmol 2-(*N*-acetoxy-*N*-acetyl)aminofluorene (AAAF) for 1 h at 37°C in a degassed solution containing 20% ethanol and 2 mM sodium citrate, pH 6.8. The reaction was stopped by removal of the excess AAAF with water-saturated ether. The AAF-modified template was purified by HPLC using a C18 column. To convert the AAF-modified template to an AF-modified template, the AAF–DNA was incubated in 1 M NaOH, 0.25 M β-mercaptoethanol for 1 h at 37°C. The reaction was stopped by neutralization of the solution with HCl. The AF-modified DNA was purified by HPLC using a C18 column.

All templates were labeled at the amino-modified C6-dT with Cy3 NHS ester (GE Healthcare) as previously described (29). Dideoxy-terminated primers were enzymatically synthesized by terminal deoxynucleotidyl transferase (TdT) (USB Affymetrix, Inc.). DNA oligonucleotide (1 nmol), 45 U TdT and 100 nmol of the appropriate dideoxy-nucleotide-5′-triphosphate were incubated in manufacturer’s reaction buffer (USB Affymetrix, Inc.) for 6 h at 37°C. Dideoxy products were HPLC purified by reverse phase chromatography on a C18 column.

The purity and structure of all DNA oligonucleotides were confirmed by Matrix Assisted Laser Desorption and Ionization Time of Flight Mass Spectrometry.

### Labeling KF

The plasmid pX5106, encoding the KF exo^−^ (D424A) gene, and the *E.coli* strain CJ376 cells were obtained from Dr C. Joyce (Yale). KF purification and labeling with Cy5 maleimide (GE Healthcare) were described previously (29). We have previously determined that KF activity is not inhibited by the Cy5 label, and only slightly inhibited by the Cy3 on the DNA during DNA synthesis at a single position eight bases beyond the Cy3 (20 bases from 5′-end) (29). Dwell time and the association rate constant of the polymerase binding to the 20mer position were in excellent agreement with ensemble values (29).

### Extension assays

KF (100 pM) was incubated with 15 nM primer–template (16mer:33mer, 1:3 ratio) in reaction buffer (50 mM Tris–HCl, pH 7.5, 10 mM MgCl_2_, 1 mM dithiothreitol (DTT) and 100 μM dNTPs) for the indicated times at 37°C. The primers were labeled with Cy3 at the 5′-end for detection. Reactions were stopped by mixing reaction aliquots with an equal volume of loading buffer (10 mM ethylenediaminetetraacetic acid, 1 mg/ml bromophenol blue, in 10 ml of formamide) and heating the samples at 80°C for 75 s. Samples were run on 20% denaturing polyacrylamide gels for ∼16 h at 800 V. Gels were scanned on a Typhoon 9210 Variable Mode Imager (GE Healthcare).

### Single-molecule assays

DNA was surface immobilized on PEGylated quartz slides via a biotin–streptavidin bridge as previously described (29–32). Reactions were carried out in 50 mM Tris–HCl, pH 7.5, 10 mM MgCl_2_, 1 mM DTT, 50 μg/ml bovine serum albumin and an oxygen scavenging system (4% wt/vol glucose, 0.04 mg/ml glucose oxidase and 0.008 mg/ml catalase) at enzyme concentrations indicated in text. Single-molecule fluorescence resonance energy transfer (smFRET) experiments used Cy5-labeled KF (20–30 nM), whereas the protein-induced fluorescence enhancement (smPIFE) experiments used unlabeled KF (0.5 nM). Data were acquired on a custom-built, prism-based total internal reflection microscope at room temperature (∼22°C) as described (31). All single-molecule experiments were carried out at ∼50 ms time resolution, and the traces were analyzed with either a five or seven point moving average. Apparent FRET efficiencies were calculated as the acceptor intensity divided by the sum of the donor and acceptor intensities. PIFE was normalized to 1.0 for the unbound DNA state; thus, increases in PIFE reflect the relative increase in Cy3 intensity on KF binding to the DNA. All single-molecule histograms were prepared from >100 individual trajectories. The binding equilibrium can be approximated by the ratio of the populations in a PIFE histogram. However, the ratio of the zero FRET peak to the higher FRET peak(s) should be considered an upper limit because inactive acceptor and unlabeled DNA polymerase will contribute to the zero FRET population even though the DNA polymerase is bound to the DNA. The errors from FRET experiments were estimated to be ± 0.02, whereas the errors for PIFE were estimated to be ± 0.1.

## RESULTS

### Carcinogenic adducts induce polymerase stalling

The effect of an adduct on DNA synthesis depends on the sequence context within which the adduct is located, the structure and orientation of the adduct in the DNA template and the properties of the DNA polymerase. To measure the degree of inhibition by either an AF-dG or AAF-dG adduct positioned in our particular primer–template system (Supplementary Table S1), we measured running start DNA synthesis on the same AF or AAF-modified DNA template sequences used in the single-molecule studies. KF fully extends the 16mer/33mer unmodified primer–template within 5 min (Figure 1b). Using a template containing an AF-dG adduct (Supplementary Table S1), we found that KF transiently stalls one base before and across the adduct (Figure 1b) and then fully extends the primer within 60 min. However, with the analogous AAF-dG-modified primer–template, the polymerase is completely blocked one base before the adduct and no evidence for extension is observed after extended incubations (Figure 1b). Similar results were obtained using a template in which the AF or AAF-dG adduct was initially located at the templating position or across from the primer terminus (Supplementary Figures S1 and S2).

The mechanisms that lead to these polymerization results can be explained by several scenarios, some of which might be generalized to other bulky adduct structures. First, it is possible that the bulky adducts induce the polymerase to dissociate from the DNA before dNTP incorporation; second, the adduct might prevent the proper alignment of the templating base in the pol site; or third, the bulky adduct could prevent the formation of a closed ternary complex by interfering with fingers closing or dNTP binding. Biochemical (33) and crystallographic (28) evidence has suggested that an AAF-dG adduct in the templating position inhibits incorporation using this latter scenario by preventing the movement of the fingers to form a closed ternary complex. However, the mechanism by which an AF-dG adduct causes a polymerase to stall near the adduct site or how an AAF-dG adduct inhibits extension remains unknown. Because of the limitations of ensemble-averaged experiments to measure dynamic processes that might distinguish these possible mechanisms, we have turned to smFRET and smPIFE methods to monitor the binding position and dynamics of individual polymerase on AF and AAF-modified DNA templates in real-time (29,30).

### Polymerase–DNA binding dynamics monitored by smFRET and smPIFE

**Figure 2.**
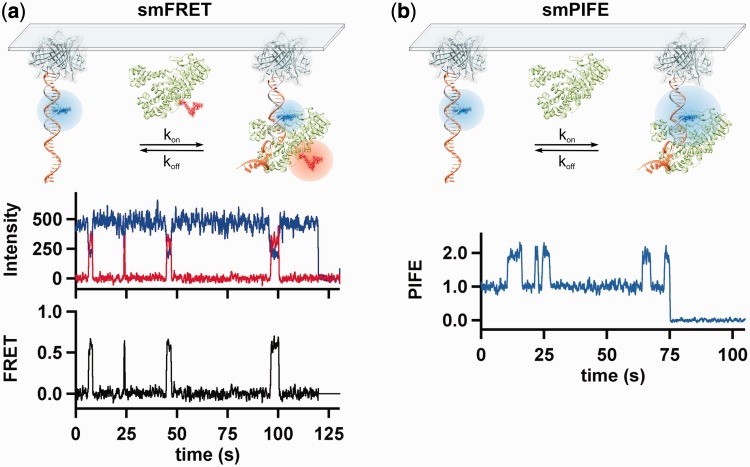
Two single-molecule approaches to monitor polymerase interactions with the DNA in real time. (**a**, top) Schematic of single-molecule FRET design. On polymerase binding to the DNA, energy is transferred from the donor Cy3 (blue) on the DNA template to the acceptor Cy5 (red) conjugated to KF. (a, bottom) Representative donor (blue) and acceptor (red) time trajectories for KF binding to the unmodified primer-template shown in Figure 3a, and the FRET trajectory (black) calculated from the donor and acceptor intensities [FRET = I_A_/(I_A_ + I_D_)]. (**b**, top) Schematic of single-molecule PIFE design. When the polymerase binds to the DNA in close proximity to the Cy3, the fluorescence intensity of the Cy3 is enhanced because of a change in the local environment about the fluorophore. (**b**, bottom) Representative PIFE trace (blue) for KF binding to the same primer–template used for (a). The Cy3 photobleached at ∼75 s.

Single-molecule approaches reveal transient events from individual molecular interactions in real-time and unveil heterogeneity within subpopulations of molecules that would otherwise remain hidden in ensemble-averaged experiments (34–36). By strategically labeling the DNA and polymerase, we can monitor polymerase binding dynamics with single-base pair resolution (29,30). In our assay, free Cy3-labeled DNA primer–template exhibits only Cy3 fluorescence (I_D_) (Figure 2a). On Cy5-labeled polymerase binding, I_D_ decreases concurrently with an anti-correlated increase in Cy5 fluorescence intensity (I_A_) (Figure 2a). The apparent FRET efficiency is calculated as FRET = I_A_/(I_A_ + I_D_) (Figure 2a). Because FRET efficiency depends on relative fluorophore distance and orientation, it reports on polymerase position and orientation on the DNA.

We have also shown that binding dynamics can be monitored with unlabeled polymerase by smPIFE (29). Polymerase binding near Cy3 changes the local fluorophore environment (viscosity and polarity), thus increasing its fluorescence quantum yield, resulting in subsequent fluorescence increases (29,37–39). We normalize PIFE to 1.0 for unbound DNA, such that relative PIFE increases report on polymerase-binding events (Figure 2b). Although smPIFE does not provide precise distance information, it offers the advantage that it is not susceptible to acceptor blinking or photobleaching, thereby providing an accurate method to measure binding kinetics (29). Using smFRET and smPIFE in tandem provides a clearer picture of polymerase dynamics on the DNA than can be obtained using either method alone.

### Templating base adducts stabilizes binary complex

**Figure 3.**
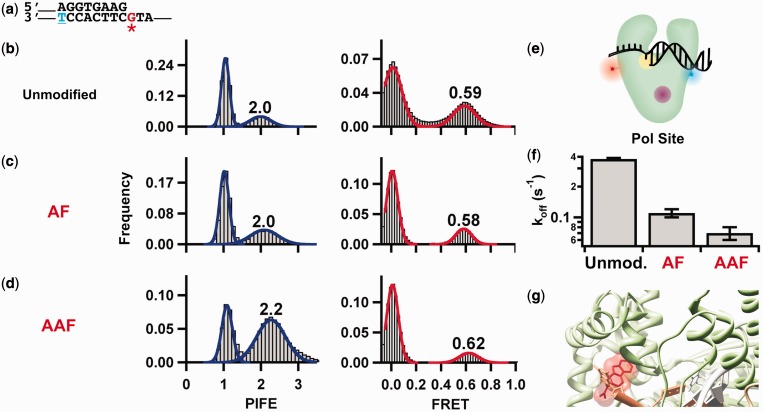
Carcinogenic adducts in ssDNA stabilize pol site binding. (**a**) Primer–template design for investigating the ensemble stalling trend observed (Figure 1b and Supplementary Figure S1) one position before the adduct. The Cy3 is conjugated to the underlined, blue thymine in the template by an amine linker. For AF and AAF lesion templates, the adduct is attached to the red guanine. (**b–d**) The PIFE and FRET efficiency histograms for KF binding to (b) unmodified, (c) AF-modified or (d) AAF-modified primer–templates. (**e**) Schematic of the primer–template bound at the pol site (yellow circle) of the DNA polymerase. During FRET, energy is transferred from the Cy3 (blue) to the Cy5 (red) dye; PIFE experiments are identical except they lack the Cy5 dye. When the polymerase binds the DNA at the pol site, the templating base and the 3′ primer terminus are within the pol site. (**f**) Comparison of polymerase dissociation rates for the unmodified, AF and AAF primer–templates. (**g**) Crystal structure with the AAF moiety intercalated into the fingers domain (PDB ID 1X9M).

To test whether bulky adducts impede polymerization by destabilizing the polymerase–DNA binary complex or by inducing polymerase misalignment, we determined binding rate constants and conformations using smFRET and smPIFE in the absence and presence of AF- and AAF-dG adducts on the templating base (Figure 3a). In the absence of an adduct, the observed FRET and PIFE values were 0.59 and 2.0, respectively (Figure 3b), in good agreement with previous results (29). The high-PIFE value indicates that Cy3, located eight nucleotides from the primer–template junction (Figure 3a), is within the molecular footprint of the polymerase. Interestingly, in the presence of either an AF or an AAF-dG adduct at the templating base, the PIFE and FRET distributions remain unchanged within experimental error (Figure 3c and d). These results indicate that neither of these bulky adducts on the templating base affect the global polymerase position or orientation on the DNA primer–template (Figure 3e).

We next used the distribution of dwell times in the high-PIFE state to determine the dissociation rate constants (k_off_, Figure 3f). In the absence of adducts, the polymerase dissociates with a rate constant k_off_ = 0.40 ± 0.01 s^−^^1^, in good agreement with previous results (29). In the presence of an AF-dG adduct, k_off_ decreases 4-fold (0.10 ± 0.01 s^−^^1^), indicating that the bulky adduct stabilizes the binary complex by 0.8 ± 0.1 kcal mol^−^^1^. In the presence of an AAF-dG adduct, the k_off_ decreases even further (0.07 ± 0.01 s^-1^), indicating that AAF stabilizes the binary complex by 1.0 ± 0.1 kcal mol^−^^1^. In agreement with our previous results (40), these data show that the bulky adducts do not induce polymerase dissociation before nucleotide incorporation, but rather stabilize the binary complex. This additional pol site stabilization and the inability to incorporate nucleotides across from the AAF adduct may be due to the intercalation of the bulky fluorene ring into the polymerase’s fingers domain, as was observed by biochemical studies (33) and in a T7 DNA polymerase co-crystal structure with a primer–template having an AAF-dG adduct located at the same position (Figure 3g) (28).

### Adducts at duplex DNA terminus induce multiple binding orientations

**Figure 4.**
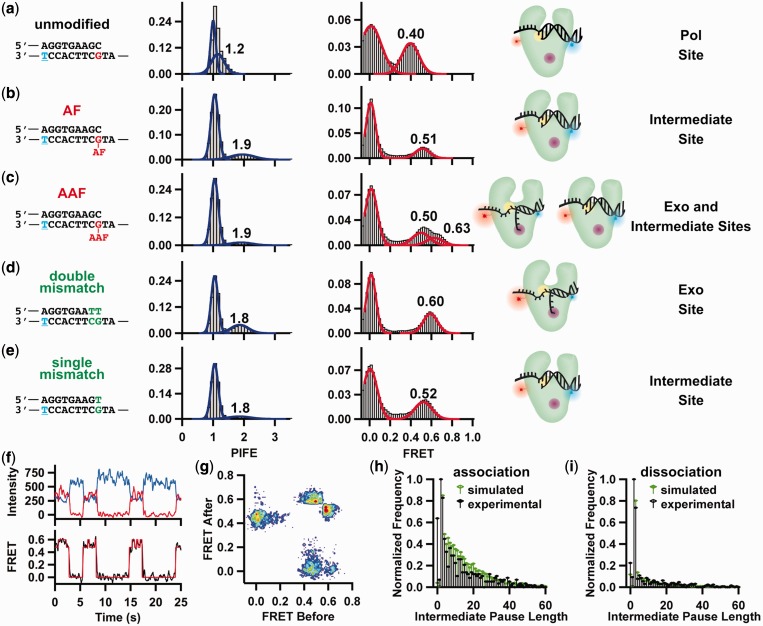
Carcinogenic adducts at duplex terminus induce distinct binding states. (**a–e**) Primer–template sequence, PIFE histogram, FRET histogram and DNA polymerase-binding states for (a) unmodified, (b) AF-modified, (c) AAF-modified, (d) double-mismatch and (e) single-mismatch DNA. Cy3 is conjugated to blue thymine. Mismatches are shown in green. For the polymerase structures, Cy3 is shown in blue, Cy5 in red, the pol site in yellow and the exo site in purple. (**f**) Representative donor (blue) and acceptor (red) time trajectories for KF binding to the AAF-modified primer–template shown in (c), and the calculated FRET trajectory (black) from the donor and acceptor intensities. The red line in the FRET trace is HMM analysis (see Supplementary Methods for details). The complete trace is part of Supplementary Figure S4b. (**g**) Transition density plot for the AAF-modified primer–template shown in (c). (**h**) Initial exo site association to an AAF-modified primer–template occurs through an obligatory intermediate state. Pause lengths measured from 10 000 simulated traces in which the intermediate site (∼0.50 FRET) was an obligatory binding step between the 0 and 0.63 FRET states (green bars) closely resemble the experimental exo site association pause lengths (black bars). (**i**) KF exo site dissociation from an AAF-modified primer–template terminus can occur directly from the exo site (60%) or through the intermediate site (40%). Simulated pause lengths for 40% dissociation from the exo site through the intermediate site and 60% direct dissociation from exo site closely fit the experimental dissociation pause lengths. See Supplementary Figure S5 and Supplementary Methods for HMM and simulation details.

We then determined the effect of positioning these adducts across from the primer terminus on polymerase-binding orientation and kinetics. On an unmodified primer–template with a primer one nucleotide longer, KF binding to the pol site yields FRET and PIFE values of 0.4 and 1.2, respectively (Figure 4a). As we have previously shown (29), this FRET value is consistent with the polymerase being one base pair further from the Cy3 donor, and the PIFE value indicates that the KF footprint no longer interacts with the Cy3. The presence of an AF-dG adduct across from a correctly paired primer terminus caused the FRET and PIFE values to increase to 0.51 and 1.9, respectively (Figure 4b). These changes in FRET and PIFE suggest that this adduct induces the polymerase to bind in an orientation different from that which occurs with an unmodified template, an orientation that causes the Cy3 to be within the polymerase footprint. In the presence of a similarly positioned AAF-dG adduct (Figure 4c), the observed PIFE value also increases to 1.9, but the FRET distribution reveals two populations centered at 0.50 and 0.63, indicating that the polymerase binds in two different orientations, one of which (0.50) is similar to that observed with the AF-dG adduct and another novel one (0.63).

Taken together, these data imply that either an AF or AAF-dG adduct at the primer–template terminus base pair prevents normal pol site binding and causes the polymerase to be positioned closer to the Cy3. These results challenge an existing crystal structure of an analogous DNA polymerase, BF, bound to AF-modified DNA at this position, which reveals no change in the polymerase-binding location relative to the duplex DNA terminus (21). A possible explanation for this discrepancy is the fact that BF lacks the 3′–5′ exo site that is present in KF (41). These alternative binding orientations may reflect the primer binding to the polymerase at a secondary position, such as at the exo site, or at a site that participates in proofreading, possibly explaining why these adducts either block or hinder DNA synthesis (Figure 1, Supplementary Figures S1 and S2) (42).

### Polymerase binds adducted DNA at either the exo or an intermediate site conformation

Previous structural and biochemical studies have identified two active sites in KF, the pol site located in the palm domain and the exo site located ∼35 Å from the pol site (43). It is possible that the altered FRET and PIFE values observed when the adduct is present in the terminal base pair could be accounted for by the transfer of the primer strand to the exo site. To test for this possibility, we compared these FRET and PIFE values with those obtained when the primer terminus contained mismatched nucleotides (Figure 4d and e). Previous studies have shown that substrates containing two terminal mismatched base pairs bind almost exclusively to the exo site of KF (29,44,45). The observed PIFE (1.8) and FRET (0.60) values with the double mismatch substrate (Figure 4d) are both significantly higher than the unmodified complementary DNA (Figure 4a), in agreement with our previous results (29). These values resemble the second population (0.63 FRET) observed with the complementary AAF-adducted primer–template (Figure 4c), suggesting that this population corresponds to exo site binding. An AAF-adducted double-mismatched primer-terminus DNA also yields similar FRET (0.63) and PIFE (1.9) values (Supplementary Figure S3), in support of this assignment. Furthermore, an AF adduct combined with the double-mismatch yields 1.7 PIFE and 0.59 FRET, in good agreement with unmodified, exo site binding (Supplementary Figure S3b), yet distinct from the complementary AF-adducted primer–template.

Using the same length primer–template, we also observed a FRET value of ∼0.50 for both the AF-dG (Figure 4b) and AAF-dG adduct (Figure 4c), a value that does not correspond to either the pol or exo KF-binding orientation. This intermediate site binding could be reproduced using a primer–template that contained a single mismatch (Figure 4e). It is possible that this intermediate state results from the rapid transfer of the primer terminus between the pol and exo sites at a rate that is faster than our time resolution (∼50 ms). This would lead to a FRET value that is the average of pol and exo orientations. We rule out this possibility because it would also yield an average PIFE value of ∼1.5, which we do not observe (Figure 4e).

One possible explanation for this intermediate site binding is that it corresponds to a distinct binding orientation that is an intermediate in the proofreading process. This assignment is supported by the single-molecule FRET trajectories for the AAF-modified primer–template, which reveal direct transitions between the intermediate site and the exo site without polymerase dissociation (Supplementary Figure S4a and b and Figure 4f). Analysis of the trajectories using a hidden Markov model (HMM) yields transition rate constants from the intermediate site to the exo site, k_exo_ = 2.1 ± 0.1 s^−^^1^, and back to the intermediate site, k_int_ = 4.3 ± 0.4 s^−^^1^. Each transition between the unbound DNA (0 FRET), intermediate site (∼0.50 FRET) and exo site (∼0.63 FRET) was counted and binned into a transition density plot (Figure 4g) (46), which reveals four major transition peaks. Two of them correspond to association (Figure 4g, 0 FRET before, 0.5 FRET after) and dissociation (0.5 FRET before, 0 FRET after) transitions to and from the intermediate site, whereas the other two (0.63 before, 0.5 after and 0.5 before, 0.63 after) correspond to shuttling between the exo and intermediate sites. The lack of prominent peaks representing direct exo site binding (0 before, 0.63 after or 0.63 before, 0 after) raises the intriguing possibility that binding of the primer terminus to the intermediate site is an obligatory step before initial binding at the exo site or dissociation from the exo site.

To distinguish between real intermediate state pausing and artifacts in the HMM analysis caused by signal averaging and exposure time integration, we carried out a series of simulations (Supplementary Methods and Supplementary Figure S5). Shown in Figure 4h is a comparison between the experimental results and a simulation for the AAF-modified primer–template in which binding to the exo site occurs through an obligatory intermediate. The fact that these simulations so closely model the experimental data supports that binding of the primer terminus to the intermediate site is an obligatory step that precedes binding to the exo site. However, for polymerase dissociation, the model best fits the experimental data when 60% of dissociations occur from the exo site and 40% from the intermediate site (Figure 4i). Sixty per cent direct dissociation is likely an upper limit because of acceptor blinking and photobleaching.

These data suggest a model for the stalling of KF when either an AF or AAF-dG is positioned across from the primer terminus. In both cases, the adduct induces the polymerase to bind away from the pol site, either into the intermediate or exo site, leading to a cessation of DNA synthesis. At that point, the models diverge for the AAF and AF adduct. The AAF-dG adduct structure apparently does not allow synthesis to continue, whereas for AF synthesis eventually continues after a delay that leads to a pause in the extension reaction (Figure 1b). The question then becomes whether extension for the AF-modified template is allowed to continue from the intermediate state or whether the presence of a dNTP causes a transition of the primer terminus to the pol site. These two scenarios can be distinguished by carrying out single-molecule experiments in the presence of the next correct dNTP.

### Correct dNTP rescues pol site binding for AF, but not AAF

**Figure 5.**
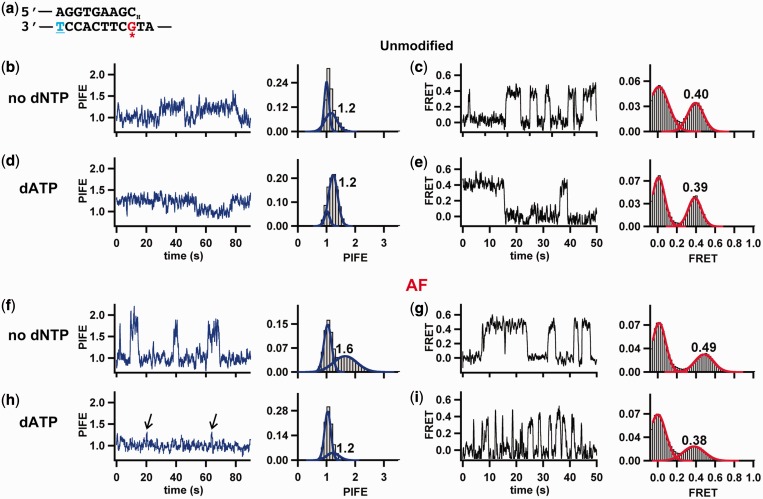
Correct dNTP rescues pol site binding for AF. (**a**) Dideoxy-terminated primer–template sequence used to observe polymerase binding to the DNA with the nucleotides present in solution. The lack of an 3′-OH in the primer prevents nucleotide incorporation. The Cy3 is conjugated to the underlined, blue thymine in the template by an amine linker. For the AF experiments, the adduct is attached to the red, asterisks guanine. (**b–i**) An example PIFE or FRET trace and the PIFE or FRET histogram for DNA polymerase binding to (**b–e**) unmodified or (**f–i**) AF-modified DNA in the absence or presence of dATP, as indicated. The PIFE and FRET states do not change on addition of the correct dNTP (dATP) for unmodified DNA; however, for AF-modified DNA, the PIFE and the FRET decrease on addition of the correct dNTP. The AF-adduct destabilizes the ternary complex ∼150-fold (*k_off_* = 2.5 ± 0.1 s^−1^) compared with the unmodified DNA ternary complex (29). The noise in our experiments can fluctuate up to ± 0.1 around a particular average FRET value. This is shown in our Gaussian distributions for each FRET histogram. Therefore, binding events at ∼0.5 FRET (Figure 5g) can fluctuate from ∼0.4 to ∼0.6. We are confident these are not transitions to the pol or exo sites for two reasons. First, the FRET distributions are clearly symmetrical and if the 0.4 or 0.6 FRET states were frequently or stably visited, we would expect shoulders on the FRET distribution (similar to Figure 4c). Second, some of the brief high FRET events observed in the trajectory are not anti-correlated transitions in the donor and acceptor intensities.

To carry out experiments in the presence of dNTPs, dideoxy-terminated primers must be used to prevent extension. Dideoxy-termination of the primers did not significantly change the observed PIFE or FRET values for the binary complex for either the unmodified or adduct-containing templates (compare Figure 4a and b, Figure 5b, c, f and g and Supplementary Figure S6b), indicating that the polymerase binds in the same pol site orientation in the absence of a 3′-OH on the primer terminus.

As we previously reported (29), addition of the next correct nucleotide (dATP in this case) does not change the PIFE or FRET values for an unmodified primer–template, indicating that the nucleotide does not change the position or orientation of the primer–template in the polymerase active site (Figure 5b and e). However, with the template containing an AF-dG adduct across from the primer terminus, both PIFE and FRET values obtained were essentially identical to those obtained using the unmodified template (Figure 5). This change was not observed with the analogous AAF-dG-modified template (Supplementary Figure S6c). These results suggest that for the template containing an AF-dG adduct, the presence of the next correct nucleotide in the active site is capable of rescuing pol-site binding by displacing the primer from the intermediate site.

The addition of an incorrectly paired nucleotide did not cause the same change for the AF-modified template as was observed when the next correct nucleotide was added (Supplementary Figure S7b). This suggests that it is the correct dNTP base pairing that triggers the conversion of the intermediate site binding to the pol site binding. Further support for this is provided by the observation that the presence of the next correct ribonucleotide (rATP) resulted in the same change as was observed for dATP (compare Figure 5h with Supplementary Figure S7c). It is interesting that KF cannot incorporate rATP in this template (Supplementary Figure S8), suggesting that the polymerase is discriminating against the ribose sugar at a subsequent step. Previous studies have shown that when an rNTP is in the active site, the closing of the fingers domain is sterically blocked by the interaction of the 2′-OH with the glutamate at position 710 (47).

**Figure 6.**
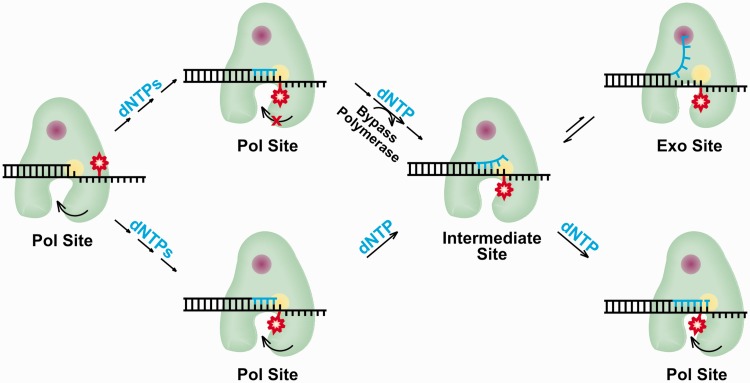
Mechanistic implications for adduct-induced distinct DNA polymerase-binding orientations. KF catalyzes template-directed DNA synthesis from the pol site (yellow) until the adduct-modified base is positioned at the polymerase active site. If bypass is prohibited, conceivably because fingers closing is inhibited as is observed with AAF-dG, polymerase action may follow the top path. In this case, a bypass polymerase would be required to add a nucleotide across from the adduct (red). If this adducted duplex terminus has a structure that still stalls the polymerase (top path), as has been found for many bulky adducts, our data suggest that nucleotide incorporation is inhibited because the adduct leads to intermediate site binding, which is in equilibrium with exonuclease site binding (purple). For an adduct that is more readily bypassed, such as AF-dG, we find that polymerase binding to the DNA follows the bottom path. Here, after incorporation opposite the adduct, the primer–template binds in the intermediate site, and the presence of the correct nucleotide rescues pol site binding allowing extension from the primer and bypass of the lesion.

Finally, we have previously shown that on unmodified templates, the correct nucleotide decelerates ternary complex dissociation by ∼10-fold compared with the binary complex alone (29,33,40). In the presence of the AF-adduct positioned across from a correctly paired primer terminus, the observed dissociation constant is *k_off_* = 2.5 ± 0.1 s^−^^1^, ∼150-fold faster than the unmodified ternary complex (29). Thus, the AF adduct destabilizes the ternary complex by ∼3.0 ± 0.3 kcal mol^−^^1^, which provides an interesting explanation for why the polymerase requires >30 min to complete elongation across AF (Figure 1b and Supplementary Figures S1 and S2).

## DISCUSSION

Gaining an understanding of how a DNA polymerase interacts with DNA adducts formed by chemical carcinogens is an important goal because these interactions are the basis for the mutagenic effects of these DNA lesions. We have used two complementary single-molecule methods, smFRET and smPIFE, to characterize these interactions for two related carcinogenic arylamine DNA adducts that we have positioned in a primer–template construct at either the templating position or across from the primer terminus. The two adducts used here represent two interesting case scenarios: one stalls polymerization completely (AAF-dG), whereas the other slows but does not block synthesis (AF-dG).

Previous studies have shown that an AAF-dG adduct inhibits DNA synthesis when located at the templating position by interfering with the closing of the fingers domain of either KF or T7 DNA polymerase (28,33), two related high-fidelity polymerases. AF-dG does not prevent the formation of the closed complex for KF (33), and presumably this represents one reason why this adduct can be bypassed by most polymerases. Much less is known about the dynamic process that occurs during the bypass of an AF-dG adduct, a process that presumably can lead to a mutation. Also unknown is the structural basis for stalling before and past the adduct location, nor how these effects compare with the AAF-dG adduct, which cannot be bypassed by KF. Even though the AAF moiety has been shown to intercalate within the fingers domain of T7 DNA polymerase when this adduct is located at the templating position (28), our data show that KF binds in the functional pol site orientation when either an AAF or an AF-dG adduct is located at this position.

However, when the primer is extended one position so that either adduct is across from the primer terminus, structures are formed that are different from that observed with unmodified DNA. For the AAF-dG case, two binding orientations are observed, one of which we identify as exo site binding and a second that has a FRET value in between that observed for pol and exo site binding. For the AF-dG adduct, only this latter intermediate binding site orientation is observed. Our data are consistent with a model in which these adducts cause the polymerase to follow one of two distinct pathways depending on the adduct structure (Figure 6). In the presence of an adduct that stalls the polymerase but eventually can be bypassed, such as AF-dG, the polymerase binds the DNA at this newly identified, non-catalytic intermediate site. Binding of a nucleotide that can correctly base pair with the templating base eventually rescues the polymerase from this state, but polymerization occurs slowly because the adducted ternary complex is destabilized ∼3.0 kcal mol^−^^1^ compared with unmodified DNA; therefore, multiple binding and dissociation cycles likely occur before phosphodiester bond formation.

In the presence of an adduct that completely stalls DNA synthesis, such as AAF-dG, our single-molecule trajectories show that after incorporation across from the adduct by a lesion bypass polymerase, the polymerase binds this structure first in the intermediate site orientation, and then the DNA is shuttled to the exo site without KF dissociation. In these studies, we used an exonuclease inactive mutant, but in a wild-type protein it is likely that the polymerase would then cleave bases from the primer 3′-end. Addition of the correct nucleotide did not rescue pol site binding, consistent with the fact that KF and other high-fidelity polymerases cannot extend primer–templates where the primer terminates across from the AAF-dG adduct. Presumably, it is structural differences between the AAF and AF adducts located in the polymerase active site and the ability of the AF-dG adduct to form a dG:dC base pair that allows the polymerase to follow the pathway that leads to extension of the primer and adduct bypass (48–50).

Future studies will test whether this mechanism applies generally to other chemical modifications and to other DNA polymerases, including those specialized in *trans*-lesion synthesis. We also plan to characterize further the intermediate binding state, which may be an intermediate in the process by which an incorrectly paired nucleotide is removed by the proofreading exonuclease activity.

## SUPPLEMENTARY DATA

Supplementary Data are available at NAR Online: Supplementary Table 1, Supplementary Figures 1–8 and Supplementary Methods.

Supplementary Data
