# Supplementary material for: Carcinogenic adducts induce distinct DNA polymerase binding orientations
Source: Nucleic Acids Res. 2013 Jun 28;41(16):7843–53. doi: 10.1093/nar/gkt554 (PMC3763543; doi:10.1093/nar/gkt554)
Supplement: Supplementary Data [file supp_41_16_7843__index.html]

Carcinogenic adducts induce distinct DNA polymerase binding orientations — Carcinogenic adducts induce distinct DNA polymerase binding orientations — Supplementary Data 

# Carcinogenic adducts induce distinct DNA polymerase binding orientations

## 

files

**Files in this Data Supplement:**

- Supplementary Data - pdf file
