# Supplementary material for: Carcinogenic adducts induce distinct DNA polymerase binding orientations
Source: Nucleic Acids Res. 2013 Jun 28;41(16):7843–53. doi: 10.1093/nar/gkt554 (PMC3763543; doi:10.1093/nar/gkt554)
Supplement: Supplementary Data [file supp_gkt554_nar-01071-d-2013-File008.pdf]

Supplementary Materials for:

Carcinogenic DNA adducts induce distinct DNA polymerase binding orientations

Kyle B. Vrtis[1], Radoslaw P. Markiewicz[1], Louis J. Romano*[,1] and David Rueda*[,1,2]

[1] *Department of Chemistry, Wayne State University, Detroit, MI, USA*

[2] *Department of Medicine, Section of Virology, Imperial College London, London, UK*

* Corresponding authors: david.rueda@imperial.ac.uk or ljr@chem.wayne.edu

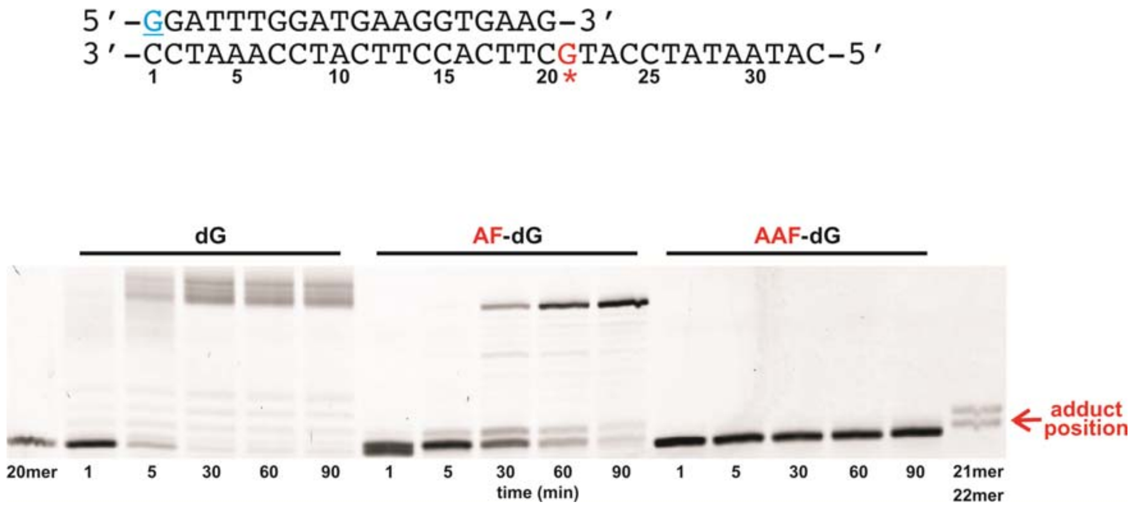

Supplementary Figure S1. DNA extension reactions on modified DNA using a standing start. The reactions were carried out identically to extension reactions shown in Figure 1 except that the primers used in these reactions terminated one base before the adduct position (20mer primer).

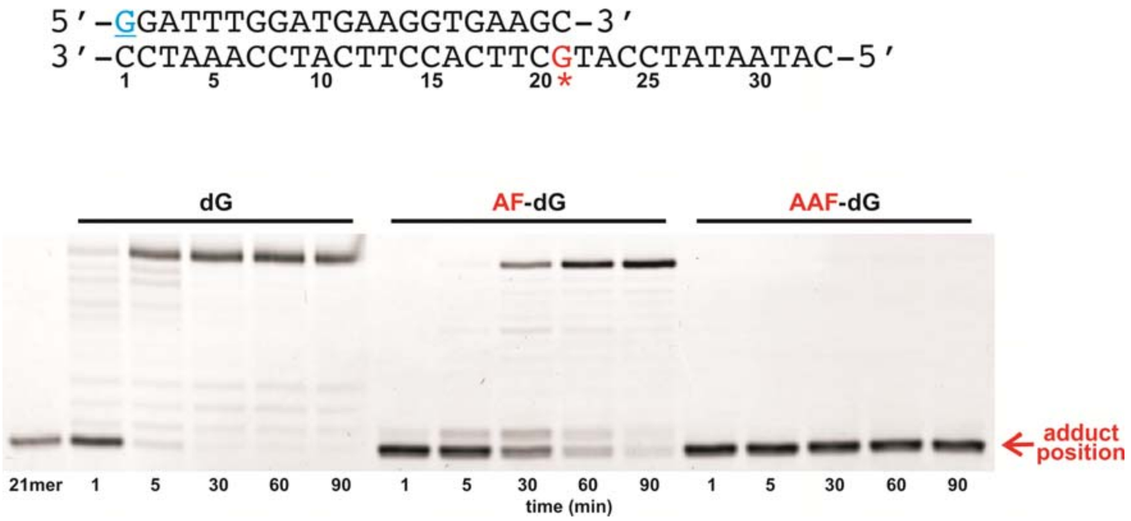

Supplementary Figure S2. Polymerase can not extend AAF primer when the adduct is at duplex DNA terminus. The standing start reactions were carried out identically to the 16mer extension shown in Figure 1; however, the initial primers used in these reactions terminated across the adduct (21mer primer). The unmodified dG primer-template and the AF-modified primer-template were nearly fully extended by 5 min and 90 min, respectively. No extension occurred on the AAF modified primer-template.

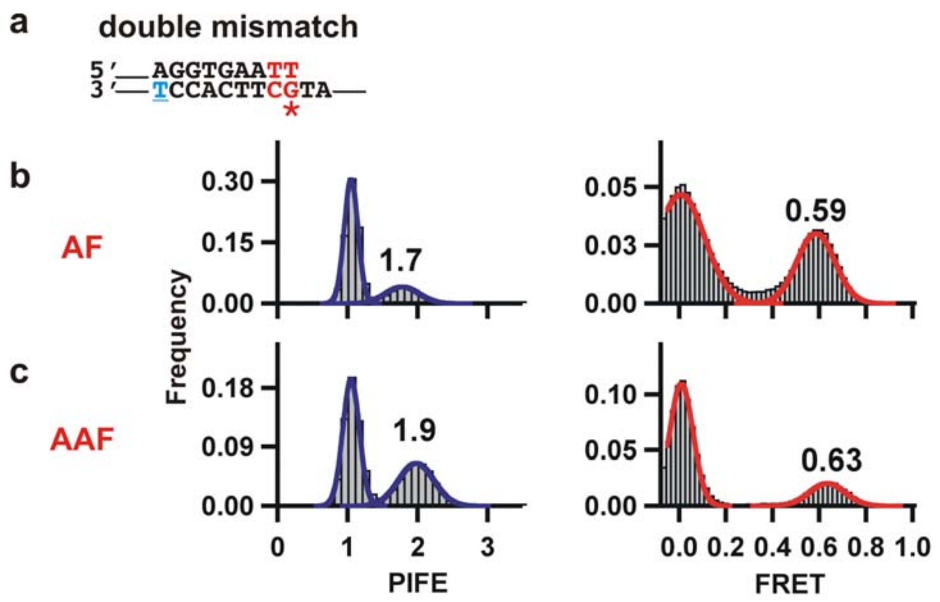

Supplementary Figure S3. A double mismatch induces exo site binding for both an AAF and AF-modified primer-template (**a**) Double mismatch primer-template sequence with the adducts at the duplex terminus. The double mismatch at the terminus was used to induce exo site binding. The Cy3 is conjugated to the underlined, blue thymine in the template by an amine linker. The adducts are attached to the red, asterisk guanine. (**b** and **c**) PIFE (left) and FRET (right) histograms for polymerase binding to (**b**) AF- or (**c**) AAF-adducted primer-templates. The AAF PIFE and FRET values match the high FRET state from Figure 4c.

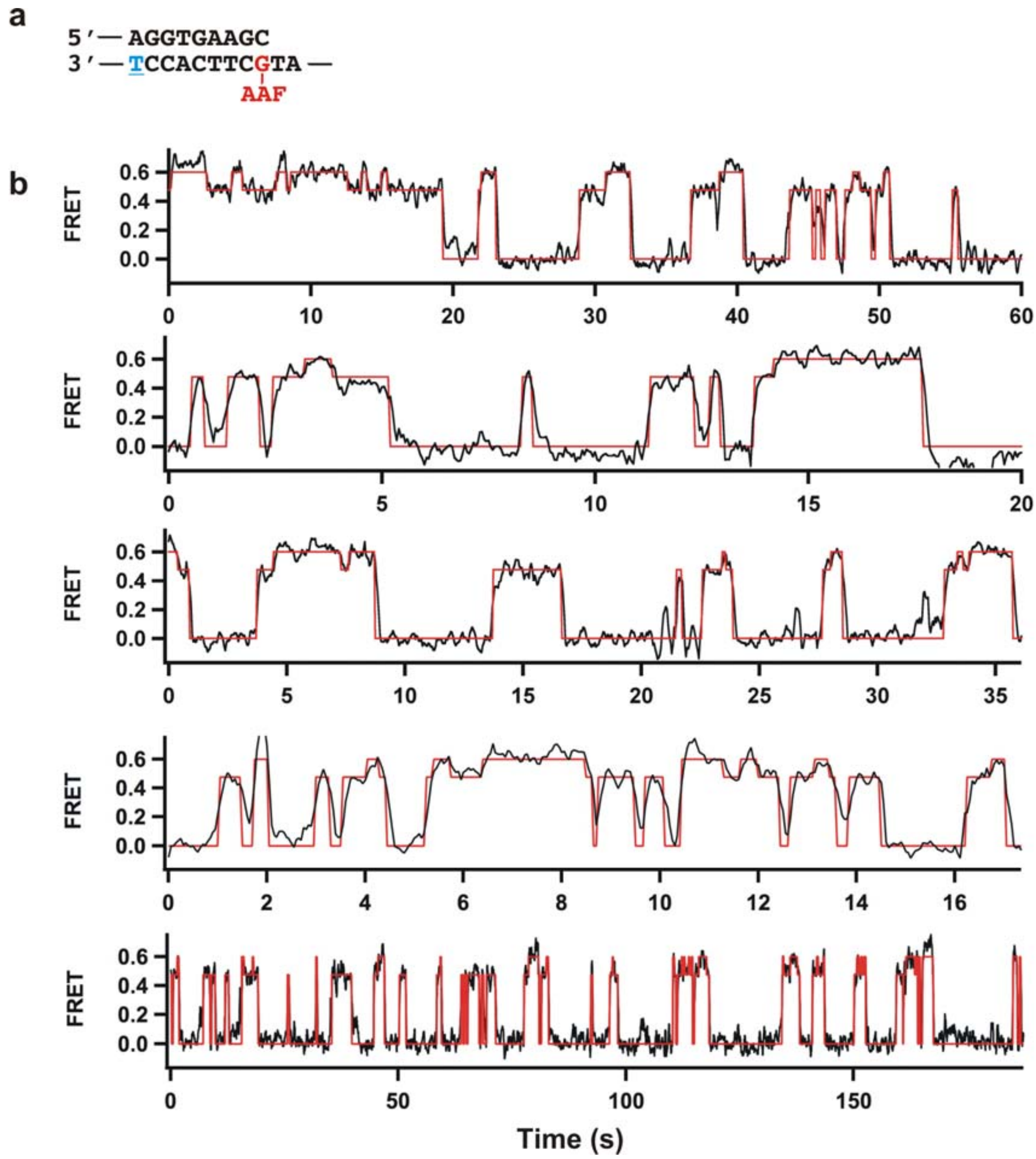

Supplementary Figure S4. KF transitions between intermediate and exo states. (**a**) AAF-modified primer-template sequence. The Cy3 is conjugated to the underlined, blue thymine in the template by an amine linker. The AAF adduct is attached to the red guanine. (**b**) Three representative FRET trajectories (black) calculated from the donor and acceptor intensities (FRET = $I_A/(I_A+I_D)$). Red lines in the traces are the FRET trajectories

calculated from HMM (see Supplementary Methods for details). The region between 135-160 s in the bottom trace is shown in Figure 4f.

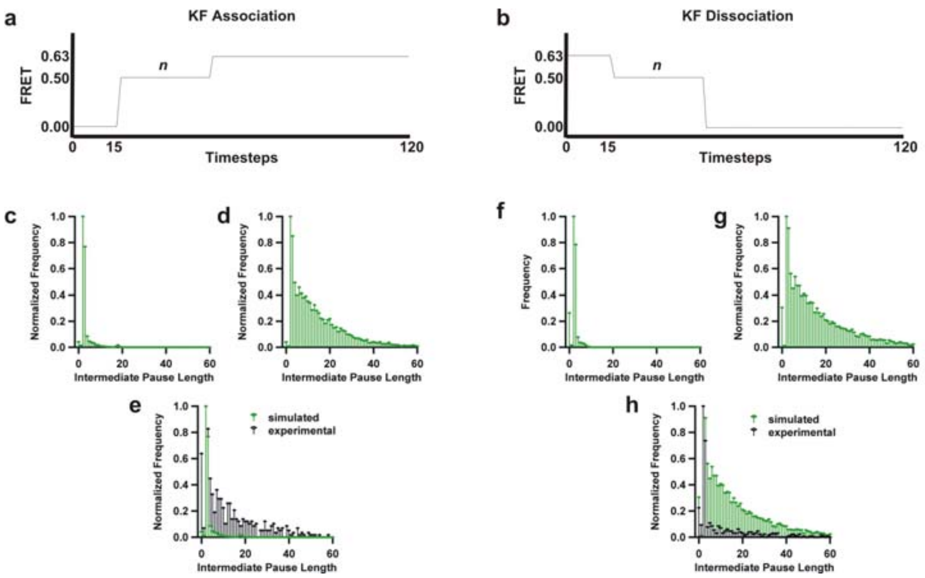

Supplementary Figure S5. Simulating intermediate site pause length for KF association to the exo site and dissociation from the exo site. (**a** and **b**) Steps used to simulate KF association to exo site and dissociation from exo site. *n* is the number of intermediate state timesteps simulated. (**c** and **f**) Simulated results from 10,000 traces for direct association to exo site or dissociation from exo site (*n* = 0). (**d** and **g**) Simulated results from 10,000 traces for association to the exo site through an obligatory intermediate or dissociation from the exo site with an obligatory intermediate. (**e**) Simulation of KF direct dissociation results from (**c**) plotted with the experimental association results. (**f**) Simulation of KF dissociation through an obligatory intermediate from (**g**) plotted with the experimental association results. See Supplemental Methods for details.

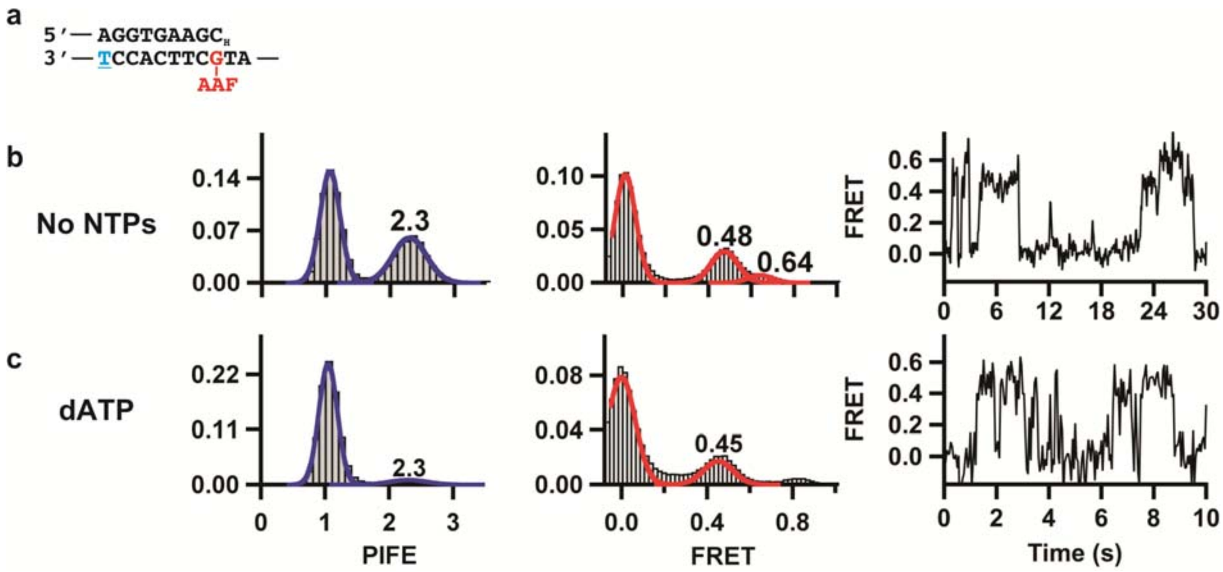

Supplementary Figure S6. AAF adduct prevents proper alignment. (**a**) Dideoxy-terminated primer-template sequence used to observe polymerase binding to the DNA with the nucleotides present in solution. The lack of the 3' OH at the primer terminus prevents nucleotide incorporation. The Cy3 is conjugated to the underlined, blue thymine in the template by an amine linker. The AAF adduct is attached to the red, guanine. (**b** and **c**) PIFE and FRET histograms for polymerase binding to the AAF-modified primer-template in the presence or absence of the correct dATP, as indicated. The 0.64 FRET state observed without NTPs is not present with dATP in solution. Additionally, a small ~0.8 FRET state is infrequently observed in the presence of dATP. Representative FRET traces for each condition are shown on the right.

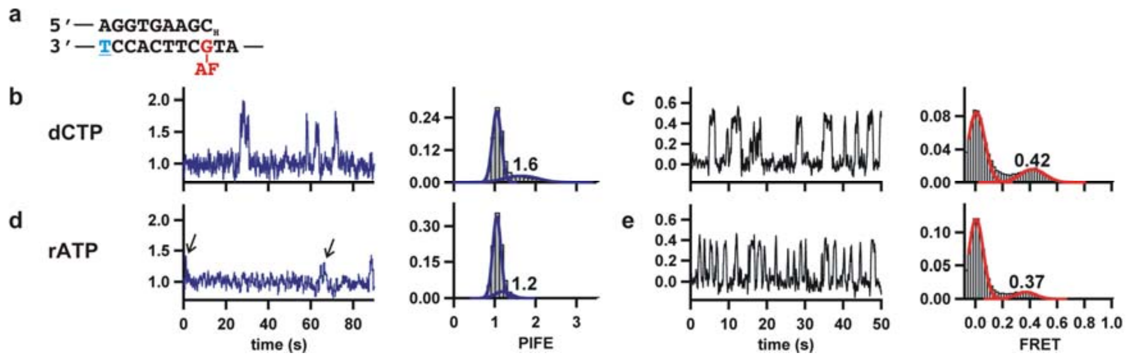

Supplementary Figure S7. Complementary base rescues pol site binding for AF. (**a**) Dideoxy-terminated primer-template sequence used to observe polymerase binding to the DNA with the nucleotides present in solution. The lack of the 3’ OH at the primer-terminus prevents nucleotide incorporation. The Cy3 is conjugated to the underlined, blue thymine in the template by an amine linker. The AF adduct is attached to the red, guanine. (**b-e**) Examples of PIFE or FRET traces and the PIFE or FRET histograms for DNA polymerase binding to AF-modified DNA in the presence of dCTP or rATP, as indicated. Interestingly, the PIFE and FRET for polymerase binding to the AF-modified primer-template in the presence of rATP decrease to values that resemble the polymerase binding to the unmodified primer-template in the presence of the correct dATP.

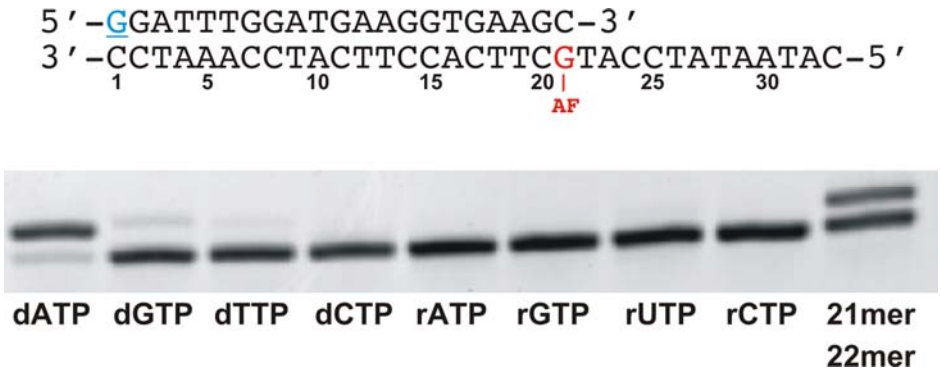

Supplementary Figure S8. KF only incorporates the correct nucleotide on an AF-modified primer. Single nucleotide incorporation reactions were carried out under identical conditions to the extension reactions (methods) except that only one nucleotide was added to the reaction, as indicated. Reactions were stopped after 60 min and run on the gel.

Supplementary Table 1. DNA sequences for ensemble and single-molecule experiments

**Extension gel assay oligonucleotides**[1]	
**16mer-Cy3 primer**	5′-[Cy3]-GGA TTT GGA TGA AGG T-3′
**20mer-Cy3 primer**	5′-[Cy3]-GGA TTT GGA TGA AGG TGA AG-3′
**21mer-Cy3 primer**	5′-[Cy3]-GGA TTT GGA TGA AGG TGA AGC-3′
**33mer template**	3′-CCT AAA CCT ACT TCC ACT TCG TAC CTA TAA TAC-5′
**Single molecule oligonucleotides**[2,3]	
**20mer-biotin primer**	5′-[Biotin]-GGA TTT GGA TGA AGG TGA AG-3′
**21mer-biotin primer**	5′-[Biotin]-GGA TTT GGA TGA AGG TGA AGC-3′
**21mer single T mm primer**	5′-[Biotin]-GGA TTT GGA TGA AGG TGA AGT-3′
**21mer double TT mm primer**	5′-[Biotin]-GGA TTT GGA TGA AGG TGA ATT-3′
**33mer-Cy3 template**	3′-CCT AAA CCT ACT TCC ACT TCG TAC CTA TAA TAC-5′

[1] Underlined red G was modified with either AF or AAF (see methods).

[2] Cy3 is linked to the underlined blue T (see methods).

[3] Green T's highlight mismatched (mm) nucleotides on the primer.

Supplemental Methods

Analysis of transitions between intermediate state and exo site by hidden Markov modeling (HMM). HMMs probabilistically determine the FRET state at a given time from defined model parameters (transition probability matrix and emission probability functions), prior state information, and the current observation (current FRET value) (1). The most probabilistic hidden path is determined using the Viterbi algorithm and the given model parameters (1).

We empirically determined the defined model parameters used as initial guesses for the HMM. As initial guesses, we used transition probabilities of 0.02 for transitions to either of the higher FRET states from 0 FRET, 0.05 for transitions to 0 FRET from either of the higher FRET states, and 0.10 for transitions between 0.5 and 0.63 FRET. The emission probabilities were calculated from normalized probability distributions centered at the three FRET states (0, 0.5, and 0.63) with 0.1 FRET standard deviations. The FRET states for each event in the TDP (Figure 4g) were determined from the mean FRET between transitions.

Simulating the obligatory intermediate state. Simulations were used to determine if intermediate state binding was an obligatory step for polymerase association to the exo site or dissociation from the exo site for an AAF-modified primer-template. All simulations were carried out with 10,000 one-binding event traces for transitions between the 0 and 0.63 FRET states. The traces consisted of the following: 15 time steps at either 0 or 0.63 FRET, followed by intermediate time steps of varying length (*n*), and finishing with 105 – *n* time steps at the post-transition FRET state (0 or 0.63) for a total trace length of 120 time steps (Supplementary Figure S5a,b). The FRET at the

given time step was randomly chosen from a normalized probability distribution centered at the given FRET state with a FRET deviation of 0.1. Each trace was averaged with a five-point moving average as was used to analyze the experimental data. The traces were analyzed by the HMM described above and the length of the intermediate FRET state (0.50) pauses determined by the HMM were enumerated.

Simulations of direct transition between the two FRET states (n = 0) usually resulted in either 2 or 3 time step intermediate pauses (Supplementary Figure S5c,f) due to averaging and exposure time integration. Therefore, the majority of the experimental intermediate pause length events 4 time steps or longer are the result of real intermediate state binding between the 0 and 0.63 FRET states. The rates for the intermediate state pauses were determined by fitting single exponential curves to the experimental data (Supplementary Figure S5e,h, black bars) 4 time steps and longer. These rates were then used to simulate obligatory intermediate state pauses between the 0 to 0.63 FRET states (Supplementary Figure S5d,g).

1. McKinney, S.A., Joo, C. and Ha, T. (2006) Analysis of single-molecule FRET trajectories using hidden Markov modeling. *Biophys J*, **91**, 1941-1951.
